# Benevolent leadership enhances organizational learning through communication, trust, and knowledge sharing

**DOI:** 10.1038/s41598-025-15170-x

**Published:** 2025-08-13

**Authors:** Salman Iqbal, Sami Ullah, Marek Zanker, Fatima Zainab

**Affiliations:** 1 Faculty of Management Sciences, University of Central Punjab, Lahore, Pakistan; 2 Faculty of Informatics and Management, University of Hradec Kralove, Hradec Kralove, Czech Republic

**Keywords:** Benevolent leadership, Open communication, Knowledge sharing, Organizational learning capability, Trust, Social exchange, Psychology, Human behaviour

## Abstract

This research addresses a significant gap in existing literature, which predominantly focuses on mainstream leadership styles, like transactional or transformational leadership. It illustrates how benevolent leadership, characterized by warmth and a paternalistic approach, can foster environments conducive to knowledge sharing and organizational learning. Utilizing a survey of 350 respondents from various knowledge-intensive organizations, the study employs structural equation modelling to analyze the data. The findings challenge the conventional belief that benevolent leadership inherently promotes a learning environment, suggesting that its effectiveness is significantly amplified when combined with trust and open communication. This study contributes to theoretical advancements by integrating social exchange theory with organizational learning, highlighting that the reciprocity triggered by benevolent leadership encourages employees to engage in positive knowledge-sharing behaviors, thereby enhancing organizational learning capabilities. For practitioners, these insights emphasize the importance of nurturing leadership styles that support trust and openness to foster a culture where knowledge is freely shared, thus enhancing the organization’s ability to adapt and innovate.

## Introduction

In an era marked by rapid technological advancements and shifting market dynamics, organizations face unprecedented challenges that demand agility and adaptability^1^. Learning is an advantage and a necessity for survival and success^2^. According to the resource-based view, only hard-to-imitate resources are a competitive advantage to the organization^3^. Organizational learning capability embodies these characteristics by fostering the continuous renewal of knowledge and competencies that competitors cannot easily replicate^4^. It helps find alternative ways to increase production efficiency and the development of beneficial relations with investors^5^. Studies consistently demonstrate that organizations with robust learning capabilities are better equipped to innovate, improve operational efficiency, and adapt to changes^6^. Koporcic^7^ found that market leaders are those companies that tend to be fast learners and use knowledge more effectively.

In the literature, some researchers emphasize the role of systems and processes^8,9^, while others focus on the culture or individual behaviors within the organization^10,11^. This divergence makes it challenging to measure organizational learning capability and compare findings across studies, thereby hampering a cohesive understanding of how it can be effectively developed and sustained^11^. While research has increasingly focused on the impact of leadership styles on organizational learning, benevolent leadership—a style characterized by personal warmth and a paternalistic approach—is less explored compared to transactional or transformational leadership^12,13^. Studies often overlook how such leadership might specifically influence learning in knowledge-intensive environments. There remains a need to understand how benevolent leadership can foster an environment conducive to knowledge sharing and learning. This research is critical for managers seeking to cultivate environments that enhance learning, for policymakers designing frameworks that support organizational learning, and for academicians expanding the theoretical and empirical understanding of how leadership influences learning capabilities.

Leadership styles directly influence an organization’s climate and culture of learning^14,15^. Leaders shape formal policies and informally influence the social norms that can foster an environment conducive to learning^5^. Benevolent leaders create a supportive and secure environment that encourages employees to engage in learning activities without fear of negative consequences for taking risks or making mistakes^16^. This psychological safety is crucial for learning, as it encourages experimentation and the exploration of new ideas. Employees are more likely to pursue learning opportunities and innovative solutions when they feel supported by leadership that cares about their personal and professional growth^17^. Thus, benevolent leadership, rooted in values of compassion and a deep commitment to the welfare of employees, fosters an environment conducive to learning and knowledge sharing^18^.

Benevolent leadership creates a culture where knowledge is not hoarded but shared freely among peers. Benevolent leaders often emphasize the importance of collective success over individual achievements, encouraging team members to exchange knowledge and collaborate effectively^16^. This collaborative environment is essential for organizational learning, as it ensures that knowledge is not siloed but is available organization-wide, leading to better decision-making and innovation^19^. Thus, knowledge, implicit or explicit, creation, retention, and transfer within the organization strengthens organizational learning capability^20^. Organizations need to exploit resources based on existing knowledge to promote the sharing of expertise from knowledgeable employees to those who lack it^21^.

Benevolent leaders empower their employees by delegating meaningful tasks and providing the necessary resources and autonomy to complete them, making them feel valued and part of the organization’s goals^22^. Shen^16^ argue that these aspects of benevolent leadership are seen as trustworthy and fair, which enhances open communication channels within the organization. Siyal^23^ suggests that trust creates an environment where employees feel psychologically safe to voice their ideas, ask questions, and make mistakes without fear of retribution. In a trusting environment, employees are more willing to share their insights and expertise with colleagues^24^. Thus, employee trust amplifies the effect of a benevolent leadership style on employees’ knowledge sharing behavior.

However, initiatives to facilitate knowledge sharing often fail due to a lack of open communication among employees^25^. Open communication creates a positive interpersonal and group dynamic where information exchange is valued and rewarded^26^. It enables teams to work cohesively, creating new knowledge and exchanging diverse perspectives^27,28^. In such an environment, the reciprocity inherent in social exchanges is enhanced, encouraging more knowledge-sharing^7^. Social exchange theory supports the idea that open communication facilitates the exchange of knowledge and amplifies its positive impacts on learning^29^. This research adopts employee communication as a moderator to amplify the effect of knowledge sharing on organizational learning.

The study contributes to the literature by empirically investigating the unique dynamics of knowledge-intensive firms, which rely heavily on their intellectual capabilities. This contribution is particularly significant as it addresses a gap in current research, which often generalizes findings across various types of organizations without considering the specific challenges and opportunities in knowledge-intensive environments. This study contributes to a deeper understanding of the specific leadership behaviors that enhance organizational learning capabilities by focusing on a leadership style characterized by personal warmth and a paternalistic approach. These contributions are novel because they link benevolent leadership directly to enhanced learning capabilities and knowledge sharing within organizations, offering practical strategies that can be implemented across various sectors to promote a more collaborative and innovative organizational culture.

## Literature review and framework

Organizations cannot compete in dynamic markets with the mere production of new products; instead, they have to develop different capabilities to utilize their valuable resources in a way that helps to produce unique products timely and cost-effectively^30^. One significant factor that affects organizational capabilities is the size and scale of the enterprise^31^. Large enterprises, with more resources at their disposal, often have dedicated teams, budgets, and infrastructure that foster organizational learning^30^. These enterprises can invest in technology, training, and knowledge management systems that promote knowledge sharing and enhance organizational learning capabilities^2,32^. In contrast, smaller organizations may lack such resources and infrastructure, which can hinder their ability to support continuous learning and innovation^33^. However, they can compensate for these limitations by adopting informal learning practices, such as peer-to-peer knowledge sharing and flexible, adaptive processes that may be more difficult to implement in larger organizations^34^. Therefore, the scale of an enterprise and the resources available to it play a crucial role in determining its ability to foster an organizational learning capability.

Organizational structure is another factor that facilitates or hinders the flow of knowledge. Flexible, decentralized structures tend to encourage knowledge sharing by reducing bureaucratic barriers and fostering informal interactions among employees^35^. In contrast, rigid, hierarchical structures may restrict knowledge flow and inhibit learning by fostering silos and reducing employee autonomy^36^. Thus, organizational learning capability is deeply intertwined with the social dynamics within an organization, particularly through internal social exchanges among employees, management, and teams^11^. These social interactions form the backbone of how information is shared, knowledge is transferred, and learning is institutionalized within the organizational framework^24^. Social exchange theory (SET) helps understand the relationship between internal social exchanges and organizational learning capability^37^. SET posits that social behavior results from an exchange process to maximize benefits and minimize costs^38^. Marshall^39^ stated that both parties have an independent relationship due to bidirectional transactions occurring in social exchange. When social interaction occurs, both parties form a sense of responsibility and obligation towards one another^40^. Therefore, when an organization demonstrates its commitment to learning and adapting, individuals perceive this as an organizational investment in the relationship, prompting them to contribute more openly to the exchange^29^.

Benevolent leadership, characterized by leaders’ goodwill and supportive behaviors towards subordinates, creates a sense of indebtedness and reciprocity among employees^18^. SET suggests that when leaders display care and generosity, employees feel a social obligation to reciprocate with positive behaviors, such as trust^39^. In SET terms, trust lowers the perceived risks of social interactions, making it more likely for employees to engage in beneficial exchanges with the leadership and within the organization^24^. Trust is a social currency that reduces the transaction costs associated with knowledge sharing^37^. When trust is present, employees are more likely to believe that their knowledge will be used appropriately and receive fair credit for their contributions^23^. The accumulation of shared knowledge due to benevolent leadership and employee trust can enhance organizational learning capability by creating a repository of information and experiences that can be drawn upon to make better decisions and innovate.

**Fig. 1:**
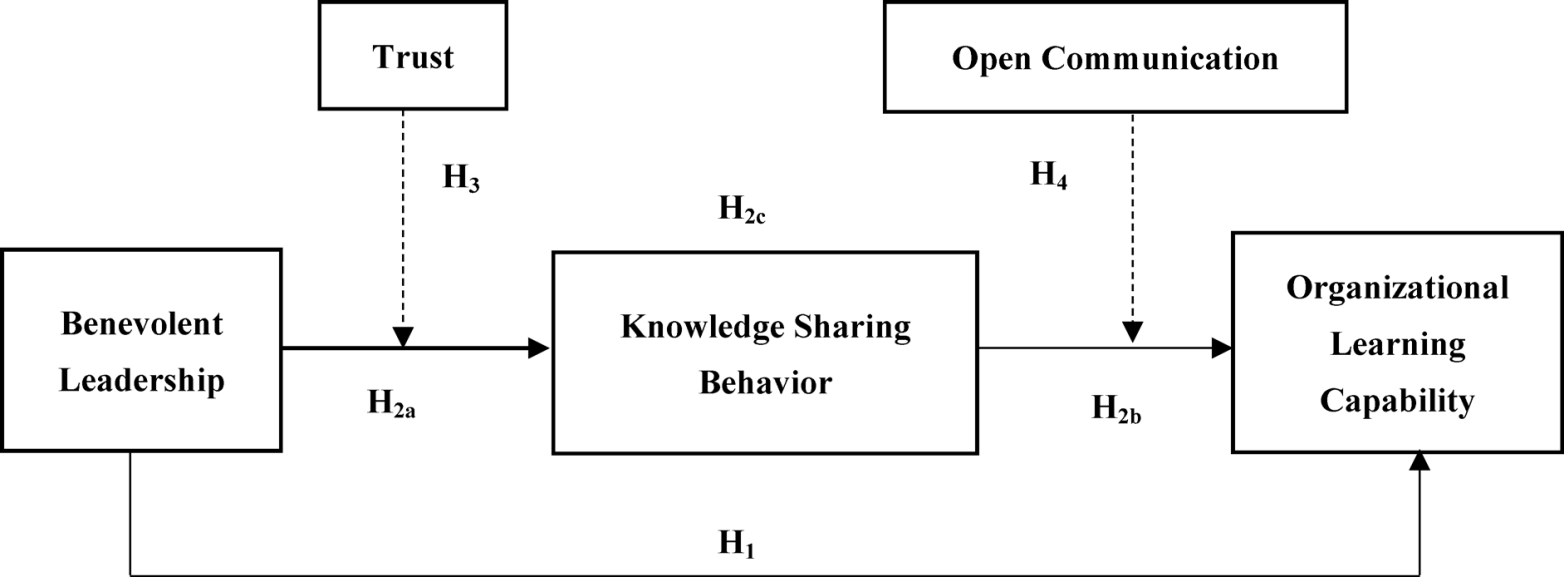
Conceptual framework of the study.* Source* Authors’ own work

Knowledge sharing is an exchange process where individuals provide valuable information to others with the expectation of future benefits^19^. This exchange is enhanced by open communication, which lowers barriers, facilitates understanding, and promotes a culture of reciprocity^41^. Open communication signals that the environment is receptive to exchanging knowledge and that the organization values and supports this behavior^33^. The cumulative effect of positive social exchanges influences the broader organizational culture^42^. Positive internal social exchanges create a culture that values learning, collaboration, and open communication^12^. This culture supports organizational learning by institutionalizing practices that encourage learning and knowledge sharing as part of everyday work processes. The conceptual model, rooted in the principles of SET, is presented in Fig. 1.

### Benevolent leadership and organizational learning capability

Extrapolating SET to organizational contexts, the interactions between leaders and employees are seen as exchanges that can yield positive relational outcomes when perceived as fair and beneficial by those involved^40^. Benevolent leadership aligns well with the principles of SET^43^. Leaders who exhibit benevolence engender strong social exchanges with their subordinates, fostering an environment of mutual respect^16^. This social capital is crucial in organizations, especially knowledge-intensive sectors where innovation and continuous learning are key^31,44^.

In knowledge-intensive firms, work often requires high levels of collaboration and continuous knowledge exchange^45,46^. These firms thrive on their ability to innovate and adapt, which heavily relies on their learning capabilities. Huang^17^ found that benevolent leadership significantly affects organizational learning capability. Soomro^47^ found that in Pakistan’s high power distance culture, the effects of leadership styles like transformational and benevolent leadership on outcomes such as job satisfaction and organizational commitment are pronounced. Benevolent leaders align with cultural expectations and enhance their employees’ willingness to share knowledge and engage in learning activities, thereby increasing the overall organizational learning capability^18^.

Moreover, benevolent leadership impacts organizational learning capability by influencing psychological safety and empowerment^23^. Employees in firms led by benevolent leaders are more likely to feel psychologically safe, which Zhou^48^ found crucial for encouraging experimentation and acquiring new knowledge—critical components of learning organizations. Empowered employees are more inclined to take risks, make decisions, and explore new ideas, all of which are essential for organizational learning and adaptability^22,43^.

The context of Pakistan, with its unique cultural nuances where respect for authority and expectations of paternalism in leadership are prevalent, further amplifies the impact of benevolent leadership^49,50^. According to Hofstede’s cultural dimensions, Pakistan scores high on power distance, indicating that organizational hierarchical structures are accepted and expected^51^. In such environments, benevolent leadership can bridge the gap between hierarchical structures and employee needs for individual attention and care, enhancing the organizational learning capability. Benevolent leadership, therefore, is instrumental in building and sustaining learning capabilities within organizations operating in culturally complex and dynamic environments like Pakistan^16,48^. Therefore, it can be proposed that:

#### H_1_

 Benevolent leadership positively influences organizational learning capability

### Mediating role of knowledge sharing

Knowledge-intensive firms rely heavily on their workforce’s intellectual capabilities and expertise, requiring robust knowledge-sharing mechanisms to maintain a competitive edge^52,53^. With its evolving economic landscape and increasing integration into global markets, Pakistan has seen a rise in such firms^23^. Here, benevolent leadership can be a key differentiator. Benevolent leadership engenders trust and psychological safety, creating an environment where employees are motivated to exchange ideas and insights freely^17,18^. When leaders demonstrate genuine concern for employees’ personal and professional development, it fosters a sense of loyalty and commitment among employees^43^. This reciprocity, a core tenet of SET, encourages employees to engage more fully in knowledge-sharing activities. Chaudhary^28^ found a positive link between benevolent leadership and knowledge-sharing behaviors. Also, Salehi and Sadeq Alanbari^33^ found that when leaders are perceived as benevolent, employees are more likely to share critical, often tacit, knowledge, which they might otherwise withhold for fear of exploitation or undervaluation.

The impact of knowledge sharing on organizational learning capability is equally significant^12^. Organizational learning capability mainly depends on the richness and depth of the knowledge shared among its members^7,54^. As knowledge is disseminated within the organization, individuals and groups synthesize this information to develop new skills, refine processes, and improve decision-making, thereby enhancing the organization’s capacity to learn and innovate^9,11,37^. Moreover, in knowledge-intensive contexts, the nature of work often requires collaboration and innovation, processes that are heavily dependent on the effective exchange of knowledge^31,45^. Thus, leaders in these firms should manage the workflow and foster an environment that promotes learning and knowledge exchange^5^. It is crucial in a country like Pakistan, as Luqman^21^ found that traditional organizational practices are more hierarchical and rigid, potentially stifling knowledge flows.

Benevolent leadership style not only promotes psychological safety but also encourages employees to engage in knowledge sharing actively and often recognizes and rewards employees who share knowledge, thereby motivating further sharing^28,55^. This recognition can manifest in various ways, including career advancement opportunities, public acknowledgement, or direct rewards, which further embed knowledge sharing into the organizational culture^36,56^. As a result, a culture of openness and collaboration is cultivated, where knowledge-sharing becomes a normative behavior^12,57^. This cultural shift is pivotal in enhancing the organization’s learning capability. Thus, benevolent leaders can substantially improve their organization’s ability to learn and adapt by fostering an environment conducive to knowledge exchange, which is vital in today’s rapidly changing business landscapes. Therefore, it can be proposed that:

#### H_2a_

Benevolent leadership positively affects knowledge sharing behavior of employees

#### H_2b_

Knowledge-sharing behavior of employees positively affects organizational learning capability

#### H_2c_

 Knowledge-sharing behavior mediates the relationship between benevolent leadership and organizational learning capability

### Moderating role of trust

According to the knowledge-based view (KBV) of the firm, knowledge is a critical organizational resource and a primary source of competitive advantage^58^. Employees often hold back valuable information for fear of criticism or undermining their unique value within the organization^33^. Trust is essential in such social exchanges^40^. Trust can be defined as faith an individual has in the intention and behavior of the other party^59^. Siyal^23^ suggested that only trusted employees share their knowledge with their peers. Bhatti^60^ found that affective trust improves knowledge-sharing behavior among executives and ultimately leads to organizational learning capability.

Benevolent leadership supports a culture of trust and openness, reducing knowledge hoarding and encouraging employees to freely share their insights and expertise, which is vital for innovation and organizational learning^5,16^. Under benevolent leadership, employees who trust their leaders are more likely to reciprocate the positive treatment they receive by engaging in helpful behaviors such as sharing knowledge, creating a virtuous cycle of knowledge sharing and collaboration^24,25,31^. Jiang and Chen^61^ argue that in environments where benevolent leadership prevails, employees will likely develop higher trust in both their leaders and the organization. When trust is high, employees are more likely to share critical knowledge, believing that their actions will lead to mutual benefits rather than personal losses.

Pakistani culture places a high value on respect and loyalty within hierarchical relationships, which can be leveraged through benevolent leadership to encourage knowledge sharing^51^. In such settings, benevolent leaders who build strong relationships based on mutual trust will likely see more robust knowledge-sharing behaviors^50^. It is especially relevant in industries where the rapid evolution of knowledge bases is critical to business success^45^. Achdiat^12^ highlighted the importance of cultural dimensions, including collectivism and power distance, which interact with leadership behaviors and trust to influence organizational practices such as knowledge sharing. Therefore, it can be proposed that:

#### H_3_

Trust positively moderates the effect of benevolent leadership on employee knowledge-sharing behavior

### Moderating role of open communication

Open communication facilitates a culture where information is freely exchanged, questions are encouraged, and feedback is constructive^42^. This environment reduces the perceived risks associated with sharing knowledge—such as loss of power or uncertainty about how the information will be received—thereby fostering an atmosphere where employees are more willing to engage in knowledge-sharing behaviors^62^. Berraies and Chouiref^45^ suggest that employees are more likely to share knowledge when their contributions are valued and that the organizational climate is supportive. It is particularly relevant in knowledge-intensive firms where the business relies heavily on its employees’ expertise and intellectual capabilities^31^. In such firms, the ability to innovate and continuously learn from internal and external sources of knowledge is essential for sustaining competitive advantage^7^.

SET postulates that when employees perceive open communication, they are likely to feel a sense of obligation that encourages the sharing of knowledge^24^. The moderating effect of open communication can be seen in how it enhances the impact of knowledge sharing on organizational learning capabilities. When open communication is prevalent, it facilitates the initial sharing of knowledge and ensures that the shared knowledge is effectively integrated into organizational processes^28,42^. This effective integration enhances learning by enabling the organization to capture, disseminate, and utilize the knowledge being shared among employees^11^. Anser^63^ found that firms in Pakistan with a culture of open communication were better able to leverage employee knowledge to improve operational efficiency and innovate product offerings. It suggests that open communication does not merely support knowledge sharing but amplifies its positive impact on organizational learning. Therefore, firms aiming to bolster their learning capabilities should foster an environment of open communication as a key strategic element. Thus, it can be proposed that:

#### H_4_

Open communication positively moderates the effect of employee knowledge-sharing behavior on organizational learning capability

## Methodology

The IT sector, specifically software development, has been identified as a crucial area for economic growth in Pakistan. The government and private sector are increasingly investing in this sector to harness its potential for export earnings and job creation. Software houses are inherently knowledge-intensive environments where innovation, problem-solving, and technical proficiency are crucial. These firms rely heavily on continuously updating and sharing technical knowledge and skills to remain competitive. Thus, the dynamics of benevolent leadership, trust, knowledge sharing, and organizational learning are particularly pronounced and crucial for success in this sector. It can guide policy and organizational decisions to maximize this burgeoning sector’s productivity and competitiveness.

### Sampling and data collection

Software houses were randomly selected from the list provided by the Pakistan Software House Association (PASHA). Project Managers, Team Leaders, Senior Developers and Technical Leads were the respondents. These individuals are key knowledge workers in software houses and can provide valuable insights into the practical aspects of the constructs used in this study. These roles were targeted due to their significant involvement in decision-making and knowledge exchange processes. The data collection was carried out over a period of two months. The questionnaire was distributed electronically via email and an online survey platform to ensure ease of access and participation. Multiple reminder emails were sent to encourage a higher response rate. In total, 430 questionnaires were sent to the identified respondents. According to Hair^64^, 336 is a statistically appropriate sample size (48 items × 7 = 336), making 430 respondents an optimal target. The response was received from 321 participants, making the response rate 75%.

### Ethical approval

for the study, including the questionnaire used for data collection, was granted by the Ethics Review Committee of the University of Central Punjab, Lahore, Pakistan. The ethics approval reference number is [UCP/ORIC/TDF/App#28/2024]. Informed consent was obtained from all participants prior to their participation in the study. This consent was provided in written form, ensuring that participants fully understood the purpose of the study, their rights, and the voluntary nature of their participation. There were no experiments in this study and all methods were carried out in accordance with relevant guidelines and regulations.

### Measurement instrument

All constructs were measured on a five-point Likert scale ranging from (1) strongly disagree to (5) strongly agree. The benevolent leadership was measured on an 11-item scale adapted from Shen^16^. Employee communication was measured on a 7-item scale adapted from Hee^27^. Trust was measured on an 8-item scale adapted from Mooradian^65^. Knowledge-sharing behavior is measured using a 9-item scale adapted from Davenport and Prusak^66^ and Gillani^67^. Organizational learning capability was measured through an 11-item scale adapted from Templeton^68^.

The scales adapted in this research were tested for reliability and validity in several studies. However, pilot testing was conducted on 30 respondents to confirm the validity and reliability of the scale. The instrument was shared with three academic experts in this area for their opinions on the face validity and understandability of the statements. After minor changes in statements, the survey was shared with 30 respondents from the industry. The pilot data analysis shows that Cronbach’s alpha values are greater than 0.70, and factor loadings for all items exceeded 0.60. Therefore, the instrument is valid and reliable for further data collection.

## Results

### Measurement model

The structural equation modelling (SEM) in SmartPLS4 was used to analyze data. In step 1 of SEM, data reliability and validity were estimated through confirmatory factor analysis. In Table 1, the values of Cronbach’s alpha 0.867 for benevolent leadership, 0.855 for communication among employees, 0.868 for knowledge-sharing behavior, 0.904 for organizational learning capability, and 0.852 for trust are higher than the criterion value of 0.70^64^. The convergent validity is measured by average variance extracted (AVE), and the values for all constructs are greater than the criterion value of 0.50^64^. Also, factor loadings are greater than the criterion value of 0.60^64^. Also, in Table 1, the square root values of AVE in the diagonal are greater than inter-construct correlations, proving the discriminant validity of the data. Thus, the data is reliable and valid for further analysis. Common method bias occurs when the data gathered from a survey or study may be biased due to the method used to collect it, such as using the same scale or method for all items. This bias can distort the true relationships between variables, potentially affecting the study’s conclusions. The Harman single-factor test showed that one factor is responsible for 31% variation in the data, which is less than 50% criterion value; therefore, common method bias is not a problem in this data. Therefore, the data is not overly influenced by a single factor (like the method of data collection), and the results can be considered to be free from common method bias.

**Table 1:** Reliability and validity statistics.* Source* Authors’ own work

Variable	Mean	Standard deviation	Cronbach’s alpha	AVE	OLC	BL	KSB	OC	Trust
OLC	4.25	0.10	0.883	0.615	**0.784**				
BL	4.08	0.06	0.830	0.514	0.215	**0.717**			
KSB	4.12	0.09	0.827	0.512	0.308	0.111	**0.716**		
OC	3.98	0.11	0.809	0.522	0.118	0.089	0.153	**0.722**	
Trust	4.21	0.08	0.781	0.533	0.101	0.162	0.415	0.385	**0.730**

The bold values in the diagonal are the square root of AVE, and these values are higher than the other interconstruct correlation values in the column, proving discriminant validity of the data.

### Structural model

**Fig. 2:**
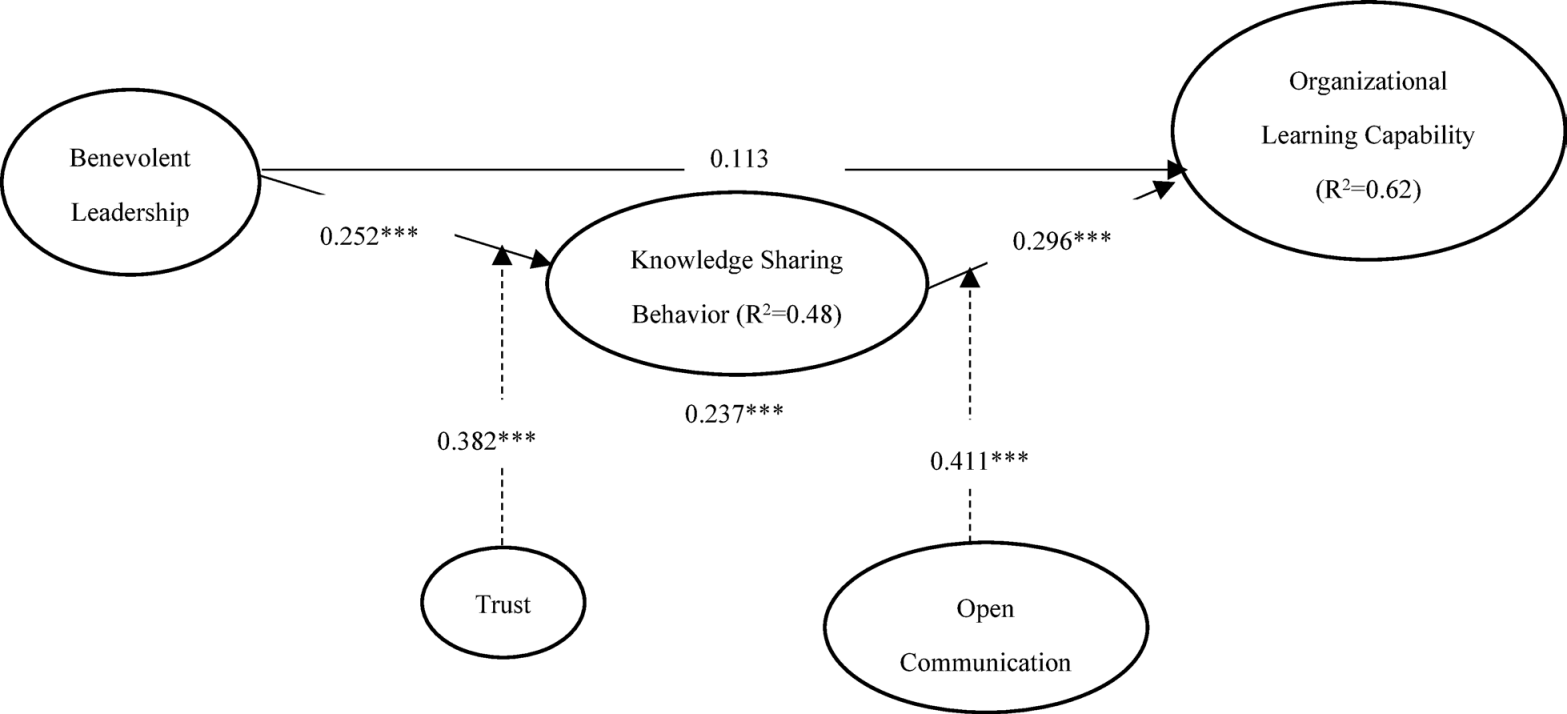
Structural path estimates in SEM.* Source* Authors’ own work.

In step 2, structural path coefficients are analyzed to test hypotheses. The values for model fit statistics, Chi-square (χ^2^=2.1), Comparative Fit Index (CFI = 0.96), Tucker-Lewis Index (TLI = 0.94), and Root Mean Square Error of Approximation (RMSEA = 0.06), indicate that the model fits the data well. The results reported in Table 2; Fig. 2 show that benevolent leadership has an insignificant effect on organizational learning capability (BL -> OLC = 0.113), rejecting H_1_. This indicates that benevolent leadership alone does not significantly contribute to organizational learning capability. However, H_2a_, examining the impact of benevolent leadership on knowledge-sharing behavior, showed a significant positive path coefficient of 0.252, suggesting a strong positive effect in favor of H_2a_. Furthermore, elaborating on the dynamics of knowledge sharing, the path coefficient (KSB -> OLC) of 0.296*** for H_2b_ confirmed the pivotal role of knowledge sharing in enhancing organizational learning.

**Table 2:** Path estimates of structural equation modelling.* Source* Authors’ own work.

Hypothesis	Path	Path coefficient	Result
H_1_	BL -> OLC	0.113	Rejected
H_2a_	BL -> KSB	0.252***	Accepted
H_2b_	KSB -> OLC	0.296***	Accepted
H_2c_	BL -> KSB -> OLC	0.237***	Accepted
H_3_	BLxTrust -> KSB	0.382***	Accepted
H_4_	KSBxOC -> OLC	0.411***	Accepted

The mediated relationship stated in H_2c_ (BL -> KSB -> OLC), where benevolent leadership impacts organizational learning capability through knowledge-sharing behavior, was also supported with a path coefficient of 0.237***. It suggests a significant indirect effect of benevolent leadership on organizational learning capability via knowledge sharing. The interaction effect of benevolent leadership and trust on knowledge-sharing behavior, resulting in a path coefficient (BLxTrust -> KSB ) of 0.382***, proves the significant moderating effect of trust proposed in H_3_. This robust effect illustrates that employee trust amplifies the positive effect of benevolent leadership on employees’ knowledge-sharing behaviors. Lastly, H_4_ tested the interaction between knowledge-sharing behavior and open communication on organizational learning capability. The model yielded a path coefficient (KSBxOC -> OLC ) of 0.411***, indicating a significant moderating effect. It confirms that knowledge sharing and open communication substantially boost organizational learning capabilities.

## Discussion

The study presents an intriguing finding: benevolent leadership alone does not significantly impact OLC. This observation challenges the prevailing view in literature that benevolent leadership inherently fosters a conducive learning environment. For instance, prior research by Huang^17^ and Shen^16^ suggests that benevolent leaders enhance employees’ engagement in learning activities by virtue of their supportive nature, thus driving OLC. However, the results from this study suggest that the direct influence of benevolent leadership on OLC may be overstated without considering other mediating variables, such as knowledge sharing. The lack of direct influence of benevolent leadership on OLC is not counterintuitive, as is the nature of work in knowledge-intensive firms. These firms often require a high degree of autonomy and expertise from employees, which might not be sufficiently stimulated by benevolent leadership’s generally supportive and paternalistic approach^45^. Also, the emphasis is more on exchanging and applying expert knowledge rather than merely providing a supportive leadership style^31^.

The results robustly support the mediation role of knowledge sharing, highlighting that benevolent leadership effectively fosters knowledge sharing, significantly enhancing organizational learning capability. This finding is well-supported by the literature, where Chaudhary^28^ and Salehi and Sadeq Alanbari^33^ emphasize the positive impact of benevolent leadership on knowledge-sharing behaviors. This mediation pathway corroborates the arguments presented by Alerasoul^11^ regarding the critical role of social dynamics and internal social exchanges in fostering organizational learning. This mediation by knowledge sharing is crucial in understanding the dynamics within knowledge-intensive environments where the sheer volume and complexity of knowledge require more than just supportive leadership to be effectively managed and utilized for learning.

The significant moderating effect of trust aligns with the insights from Zhang^4^ and Jiang and Chen^61^, who noted that trust enhances the willingness to share knowledge, reducing the perceived risks in social exchanges. Similarly, the strong moderating role of open communication in enhancing the effect of knowledge sharing on organizational learning capability supports the theoretical propositions by Yue^42^ and Anser^63^ about the importance of open communication in promoting a culture conducive to knowledge sharing and organizational learning. These factors suggest a more nuanced mechanism where benevolent leadership contributes to OLC through knowledge sharing fostered by trust and facilitating open communication.

This research is conducted in the context of a high-power distance culture, like Pakistan, and it is critical to understand how these findings would be different in low-power distance cultures. Benevolent leaders in high-power distance cultures are seen as more trustworthy and caring, and therefore improve knowledge sharing and organizational learning. This is supported by Soomro^47^, who suggest that paternalistic styles of leadership are especially effective in high power distance cultures where workers appreciate the paternal protection from their leaders. Similarly, Shen^16^ and Huang^17^ indicate that benevolent leadership promotes obligation and loyalty among subordinates working within high-power distance cultures, promoting knowledge sharing and teamwork. Conversely, in low-power distance cultures, effective leadership is often associated with more egalitarian leadership styles, in which leaders need to be facilitators and partners instead of paternalistic figures. Therefore, as suggested by Chan^43^, employees in low-power distance cultures might not see benevolent leadership as a prerequisite to knowledge sharing or organizational learning. Hence, it is suggested that research in the future builds on these results to investigate the role of benevolent leadership in low-power distance and less collectivist societies.

## Conclusion

This research demonstrates that while benevolent leadership alone may not directly enhance organizational learning capability, it significantly influences knowledge-sharing behaviors, substantially improving organizational learning outcomes. This relationship is further reinforced by the presence of trust and facilitated through open communication channels, underscoring the intricate interdependencies within organizational dynamics. The results highlight that in environments where leaders exhibit a supportive, trustworthy, and communicative style, there is a notable increase in employee knowledge exchange, leading to richer organizational learning and innovation. This study deepens the understanding of the mechanisms through which leadership styles impact organizational capabilities and provides actionable insights for leaders aiming to cultivate a learning-oriented organizational culture. Emphasizing the necessity for benevolent leadership in today’s rapidly evolving market landscapes, our research encourages organizations to foster leadership styles that nurture trust and open communication, ensuring that knowledge sharing becomes a cornerstone of their strategic operations.

## Implications

### Theoretical implications

Social Exchange Theory (SET) provides a framework that helps us understand how social behaviors, such as knowledge sharing, emerge from reciprocal exchanges between individuals. It suggests that social behaviors are driven by an exchange process in which individuals aim to maximize benefits and minimize costs. In this study, benevolent leadership is positioned as a key enabler of knowledge sharing within organizations^18^. The warmth and support provided by leaders foster a reciprocal relationship, where employees feel valued and, in turn, are more likely to share their knowledge, skills, and expertise. This study deepens our understanding of SET by applying it to leadership dynamics and organizational learning, shedding light on how these interactions influence both individual and collective knowledge development.

Moreover, the study also contributes to theoretical understanding by showing how benevolent leadership influences organizational learning, a process crucial for adapting to changing environments and gaining competitive advantages. It is widely recognized that knowledge sharing plays a critical role in organizational learning, as it allows employees to pool their knowledge and create innovative solutions. The study highlights that benevolent leadership can foster this sharing by cultivating trust, openness, and positive relationships. This finding builds on existing research that emphasizes the importance of trust in facilitating knowledge exchange and organizational learning through open communication^5,16,22^. The study demonstrates that the effects of benevolent leadership go beyond simple dyadic exchanges between leaders and employees. Instead, these exchanges create a broader organizational context where knowledge sharing and learning can flourish. The findings suggest that organizations that prioritize benevolent leadership may experience enhanced adaptability and innovation, as employees feel more supported and motivated to contribute to the organization’s knowledge base.

### Practical implications

From a practical standpoint, the findings of this study offer valuable insights for organizational leaders and policymakers aiming to enhance learning capabilities within knowledge-intensive firms. The apparent linkage between benevolent leadership and improved knowledge-sharing behaviors underscores the importance of adopting leadership styles that are not only supportive but also trust-building and communicative^5,16,22^. Organizations should consider training programs focusing on developing leaders who embody these qualities, fostering an atmosphere where employees feel valued, supported, and motivated to share knowledge. A Pakistani manufacturing company, ENGRO, one of its key businesses is Fertilizers, promotes employee engagement through open communication, regular feedback sessions, and leadership training. Similarly, Telenor Pakistan encourages open-door policies and internal communication platforms to connect teams. This increases collaboration and reduces workplace conflict.

However, many Pakistani companies still follow traditional, top-down management styles with high power distance^49^. This creates fear and a lack of trust between managers and staff. To improve, companies can apply benevolent leadership by listening to employee concerns, supporting work-life balance, and treating workers with kindness. When employees feel safe and respected, they are more loyal, creative, and productive. Therefore, policymakers need to understand the value of promoting leadership development and organizational learning as part of business strategy. Encouraging businesses to adopt benevolent leadership and invest in communication infrastructure can create a more collaborative, innovative environment. Ultimately, these strategies foster a culture that encourages knowledge sharing, which is essential for long-term business success in today’s competitive and fast-evolving market.

## Future research directions

Future research could explore several promising directions further to understand the dynamics of leadership and organizational learning. One significant area involves examining the scalability of benevolent leadership effects across different cultural and organizational contexts and, particularly to discern if the positive impacts on knowledge sharing and learning capabilities observed in knowledge-intensive firms hold in less knowledge-driven industries or different geographical regions. Specifically, studies could investigate how large and small enterprises implement organizational learning practices differently, and how these practices impact their ability to innovate and adapt to changing environments. Additionally, it would be insightful to investigate the long-term impacts of benevolent leadership on organizational performance metrics such as innovation rates, employee retention, and financial performance. Research could also expand on the role of technology in enhancing the effectiveness of benevolent leadership, exploring how digital tools and platforms might amplify or alter the traditional dynamics of trust and communication within firms. These directions promise to enrich academic discourse and offer practical insights for enhancing organizational strategies and leadership development programs.

## Data Availability

The datasets generated during and/or analyzed during the current study are available from the corresponding author upon reasonable request.
